# Evaluating Dental Fear and Anxiety in Pediatric Patients Visiting a Private and a Public Dental Hospital in Lahore, Pakistan

**DOI:** 10.7759/cureus.35243

**Published:** 2023-02-20

**Authors:** Talha Mobin, Tooba Zahid Khan, Anma Mobin, Muhammad R Tahir, Qirat Imran, Syed Aun M Gardezi, Rafey Waqar, Mahnoor Hanif, Mohamed Zakee Mohamed Jiffry, Mohammad A Ahmed-Khan

**Affiliations:** 1 Dentistry, CMH (Combined Military Hospital ) Lahore Medical College and Institute of Dentistry, Lahore, PAK; 2 Dornsife School of Public Health, Drexel University, Philadelphia, USA; 3 Dentistry, CMH (Combined Military Hospital) Lahore Medical College and Institute of Dentistry, Lahore, PAK; 4 Dentistry, Akhtar Saeed Medical and Dental College, Lahore, PAK; 5 Medicine and Surgery, CMH (Combined Military Hospital) Lahore Medical College and Institute of Dentistry, Lahore, PAK; 6 Internal Medicine, Danbury Hospital, Danbury, USA; 7 Internal Medicine, Danbury Hospital - Yale School of Medicine, Danbury, USA

**Keywords:** pediatrics, procedural anxiety, anxiety, dental fear, cfss-ds

## Abstract

Background

One of the biggest hurdles in treating pediatric patients is managing dental fear and anxiety. Some factors that contribute to an increase in dental anxiety are fear of pain, the presence of unknown individuals, a change in the setting of an environment, and separation from parents.

Aim

The aim of this study was to evaluate dental fear and anxiety in pediatric patients, between the ages of 6 and 12 years, visiting private and public dental hospitals using the Children’s Fear Survey Schedule-Dental Subscale (CFSS-DS).

Methods

A total of 280 children, 140 in a private dental hospital setting and 140 in a public dental hospital setting, were enrolled in this study. The purpose of the study was explained to the accompanying guardian of the patient and written consent was taken. The CFSS-DS was explained verbally in Urdu and the questionnaire was given to guardians alongside the patients which they were asked to fill out following their dental treatment.

Result

The data obtained from the questionnaires were analyzed using the unpaired t-test. The highest dental fear mean scores and standard deviation in a private dental hospital were for “choking” (3.25 ± 1.21), “the noise of the dentist drilling” (3.24 ± 1.04), and “having somebody put instruments in your mouth” (3.19 ± 1.06), whereas, for a public dental hospital, the highest fear score was recorded in “choking” (3.17 ± 1.69), “injections” (3.07 ± 1.72), and “people in white uniforms” (1.90 ± 1.21).

Conclusion

The study showed a higher prevalence of dental fear and anxiety in a private dental setting when compared to a public dental setting. Factors responsible for an increase in dental fear need to be assessed for each patient and then treatment given accordingly.

## Introduction

Dental fear and anxiety is a major challenge faced by dental practitioners all around the world, especially when it comes to treating children. The term dental fear refers to unpleasant emotions evoked in response to certain stimuli occurring in situations linked with dental treatment. Dental anxiety, on the other hand, refers to the excessive and unreasonable adverse emotions experienced by dental patients in regard to or in response to their dental treatment [1]. The term used to refer to the most extreme or heightened negative emotional response is a dental phobia, which is characterized by persistent anxiety in relation either to clearly marked situations/objects (e.g. drilling, injections) or to the dental situation in general [2]. There are many factors that contribute to eliciting heightened emotional responses in patients seeking dental treatment. The focus of this study is to comprehend the factors associated with dental fear and to understand the differing levels of dental anxiety faced by pediatric patients seeking dental treatment in private and public dental clinics.

It is believed that different clinical settings have a distinct impact on children’s behavior. In public hospitals, the environment that children find themselves in is often loud and chaotic, punctuated by dull walls, malfunctioning units, a vast number of people present during the procedure, and an overwhelming inflow of patients leading to a long waiting period and a gradual increase in anxiety levels. Along with that, the attitude of the dentist and the noise that accompanies surgical machinery only add to the child's anxieties. According to different studies, there is a strong correlation between persistent dental fear and the unwelcoming clinical attitude of dentists [3,4]. It is important to understand that a personal experience of discomfort at the dentist’s office is not the only contributing factor to dental fear and anxiety in children. Even an indirect or secondhand experience narrated to the child jokingly can produce a negative effect and cause anxiety in the future because it builds an association between dental treatment and pain in the child's mind [5].

Specifically, in the Pakistani population, the pediatric patients seeking dental care, the attitude of parents toward their children's oral health is not given much importance due to a lack of awareness [6]. Several studies have shown a link between parental fear and anxiety regarding dental treatment and their children who exhibit the same dental fear and anxiety [7,8]. Culture is most likely to influence dental fear and anxiety but individual familial practices surrounding health and health care play a similar role [9]. Along with this, there is the added factor of the treatment cost. Most of the treatment for dental problems costs a hefty sum in private clinics and setups, meaning that people from low-income families will seek treatment in government facilities. Public dental hospitals charge minimally but are less likely to provide a positive experience for their patients, especially young children when compared to a private dental setting. When these children grow up, they are likely to avoid their dental treatments, having come to associate it over time with noise, pain, and discomfort. It has been proven that adults with lower oral health literacy are less likely to seek dental care for their children [10]. The relationship between dental anxiety and socioeconomic status in children belonging to low-income families is of potential clinical importance [11].

As has been discussed already, the past dental experiences of children determine their outlook regarding their future dental treatment. A history of positive or at the very least neutral dental experiences may help to combat the development of traumatic associations or experiences, and subsequently against the acquisition of higher fears [12].

Although there are a variety of factors that contribute to the development of these negative associations, they can primarily be categorized into three categories: the personal (age, gender, general fear, temperament, intellect), the social (parental dental anxiety, family social-economic status, pre-appointment preparation by parents and their expectations for children’s behavior in dental environment), and the clinical environment (factors associated with a dental visit, treatment, environment) [13].

Dental fear scales have been widely used to assess the level of dental fear in children. The first dental fear survey was established by Scherer and Nakamura. It consisted of 80 items that had a five-point scale. Even though it showed high validity and reliability, the difficult nature of the questionnaire limited its use in child patients [14]. To overcome this limitation, The Dental Subscale of Children's Fear Survey Schedule (CFSS-DS) was developed by Cuthbert and Melamed [15].

The aim of this cross-sectional study was to assess dental fear in children between the ages of 6 and 12 years and to assess whether dental fear and anxiety differed across healthcare settings.

## Materials and methods

Study design and participant recruitment

This was a cross-sectional descriptive study that was completed in two different healthcare settings. A survey was conducted that assessed dental fear and anxiety in pediatric patients aged 6-12 years. This age group was selected because, in both dental hospitals, pediatric patients were considered up to the age of 12. A total of 280 participants were selected randomly. A total number of 140 participants were selected from CMH Institute of Dentistry Lahore, which is a private dental hospital, and another 140 participants from de Montmorency, which is a public dental hospital in Lahore. Before the start of the study, ethical approval and permission were obtained from the proper authorities.

A close-ended questionnaire was used for this study. The questionnaire contained information such as demographics and whether or not the patient had received any dental care prior to this study ever, alongside the dental fear and anxiety question tools. The inclusion criteria were pediatric patients between the ages of 6 and 12 years, with no underlying medical conditions or genetic abnormalities. Special needs children were excluded from this study as well as patients who required treatment under general anesthesia.

To assess dental fear and anxiety, a very famous scale developed by Cuthbert and Melamed, known as the CFSS-DS, was used for this study. The scale has been translated into many languages globally and is available to be used.

Survey instrument

The CFSS-DS consists of 15 questions. The answer to each question is based on a five-point Likert scale. The participants had to choose one of the following options for each question:

1 = Not afraid at all

2 = A little afraid

3 = A fair amount of afraid

4 = Pretty much afraid

5 = Very afraid

**Figure 1:**
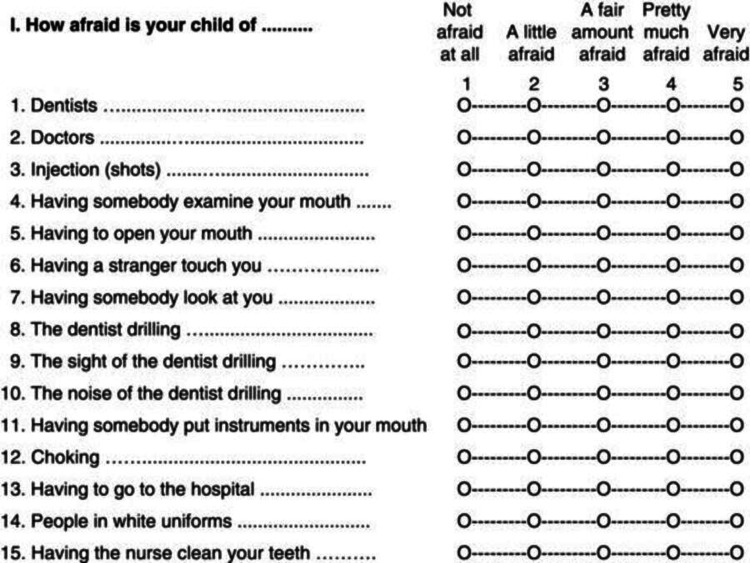
CFSS-DS CFSS-DS, Children Fear Survey Schedule-Dental Subscale.

The maximum score that can be achieved on this scale is 75 and a minimum of 15. A score of 38 or above indicates the presence of dental fear and anxiety. Figure 1 shows a diagram of the CFFS-DS.

Statistical analysis

SPSS version 26.0 (IBM Corp, Armonk, NY) was used as the statistical software for analysis. Gender and individual response to each question was expressed as a mean with standard deviation. The data were found to be normally distributed using the Shapiro-Wilk test. An unpaired student's t-test was used to assess whether there was a statistically significant difference in dental fear scores when comparing a private healthcare setting to a public healthcare setting. A p-value of less than 0.05 was considered significant.

## Results

A total of 280 children between the ages of 6 and 12 years provided complete data for each of the questions on the survey. There were 141 males (50.4%) and 139 females (49.6%). The proportion of children in each category of age is presented in Table 1. Out of the 280 children, 185 (66%) of them reported having a score of 38 or above, categorizing them as having dental fear and anxiety. Of the 185 children, 106 (57.3%) were females. There was a statistically significant difference in males (65.3%) who had a CFSS-DS score of less than 38 when compared to females (34.7%; p = 0.004), as shown in Table 2. Even though males were less afraid compared to females, both were most afraid of item number 12, “choking,” which had a mean score of 3.00 ± 1.51 in males and 3.42 ± 1.39 in females. This is depicted in Table 3. When comparing a private dental hospital to a public dental hospital about dental fear and anxiety, it was found that 63 children (34.1%) had a score of 38 or above in a public healthcare setting whereas 122 children (65.9%) had a score of 38 or above in a private healthcare setting. This was a statistically significant result as it had a p-value of 0.007. The results are given in Table 4. When comparing individual item responses in a public vs private healthcare setting, children in a private setting and public setting were most afraid of item number 12, “choking,” 3.25 ± 1.21 and 3.17 ± 1.69, respectively. The results are given in Table 5.

**Table 1:** Total number of participants of each age.

Age (years)	Males	Females	Total	Percent
6	14	12	26	9.3
7	31	18	49	17.5
8	23	23	46	16.4
9	16	39	55	19.6
10	25	17	42	15.0
11	12	18	30	10.7
12	20	12	32	11.4

**Table 2:** Dental fear and its relation to gender. CFSS-DS, Children’s Fear Survey Schedule-Dental Subscale. *Significant p-value

CFSS-DS score	Males (%)	Females (%)	t-Test value	p-Value
≥38	79 (42.7)	106 (57.3)	1.478	0.072
<38	62 (65.3)	33 (34.7)	2.756	0.004*
Total	141	139	-	-

**Table 3:** Mean score and standard deviation per question stratified by sex. SD, standard deviation. *Significant p-value.

Question number	Males (mean ± SD)	Females (mean ± SD)	p-Value
1	2.43 (1.26)	3.01 (1.46)	0.001*
2	2.29 (1.26)	2.72 (1.44)	0.009*
3	2.65 (1.46)	3.06 (1.47)	0.019*
4	2.09 (1.26)	2.57 (1.35)	0.002*
5	2.34 (1.22)	2.75 (1.31)	0.007*
6	2.40 (1.31)	2.76 (1.19)	0.02*
7	2.12 (1.17)	2.50 (1.21)	0.009*
8	2.57 (1.25)	3.09 (1.38)	0.001*
9	2.36 (1.24)	2.80 (1.36)	0.005*
10	2.68 (1.35)	3.18 (1.31)	0.002*
11	2.61 (1.33)	2.91 (1.37)	0.067
12	3.00 (1.51)	3.42 (1.39)	0.015*
13	2.48 (1.35)	2.64 (1.29)	0.296
14	2.26 (1.31)	2.33 (1.19)	0.648
15	1.96 (1.07)	2.28 (1.23)	0.022*

**Table 4:** Dental fear and its relation to the healthcare setting. CFSS-DS, Children’s Fear Survey Schedule-Dental Subscale. *Significant p-value.

CFSS-DS score	Public setting (%)	Private setting (%)	Total	t-Test value	p-Value
≥38	63 (34.1)	122 (65.9)	185	2.497	0.007*
<38	77 (81.1)	18 (18.9)	95	6.148	0.001*

**Table 5:** Mean scores and standard deviation per question stratified by the healthcare setting. SD, standard deviation. *Significant p-value.

Question number	Private (mean ± SD)	Public (mean ± SD)	p-Value
1	2.93 (1.15)	2.51 (1.58)	0.011*
2	2.94 (1.20)	2.07 (1.40)	0.001*
3	2.63 (1.15)	3.07 (1.72)	0.012*
4	2.87 (1.21)	1.78 (1.21)	0.001*
5	2.91 (1.10)	2.17 (1.34)	0.001*
6	3.04 (1.17)	2.12 (1.19)	0.001*
7	2.54 (1.15)	2.08 (1.21)	0.001*
8	2.96 (1.10)	2.70 (1.54)	0.109
9	2.71 (1.23)	2.44 (1.40)	0.085
10	3.24 (1.04)	2.62 (1.55)	0.001*
11	3.19 (1.06)	2.32 (1.48)	0.001*
12	3.25 (1.21)	3.17 (1.69)	0.655
13	3.01 (1.10)	2.11 (1.37)	0.001*
14	2.69 (1.18)	1.90 (1.21)	0.001*
15	2.43 (1.08)	1.81 (1.16)	0.001*

## Discussion

Dental fear and anxiety in children can have deleterious effects on a child’s oral health [16]. This fear can then persist in adolescence, which can lead to disruptive and neglectful behavior during dental treatment or avoidance of dental treatment at all. Furthermore, treating anxious patients then becomes more time-consuming and more stressful. That is why it is important to diagnose dental anxiety as early as possible and manage it according to the appropriate methods [17]. To diagnose dental fear and anxiety, the CFSS-DS was used, which was developed by Cuthbert and Melamed in 1982 [18]. Many studies have used this scale and their results indicate its high reliability and validity [19,20]. Many other scales such as the Dental Fear Schedule Subscale-Short Form (DFSS-SF), Dental Anxiety Scale (DAS), and Modified Corah Dental Anxiety Scale (MDAS) were also invented to measure dental fear and anxiety, but the CFSS-DS is the most widely used because it was specifically developed with a theoretical model [21]. In this study, 66% of children were diagnosed with dental fear and anxiety, out of which 57.3% were females. These results were similar to a study done by Klinberg et al., which showed that girls tended to be a bit more anxious than boys. Another study done by Shim Y.S. et al. also indicated a similar result [22]. A study done at the University of Toronto stated that even though it might be true that females are more afraid of dental treatment than males, males tend to be dishonest toward their feelings and yet are the first ones to faint in a dental office [23]. Despite gender being an associative risk factor, both sexes were most afraid of “Choking,” “The noise of the dentist drilling,” and “injections.” These results are similar to a study done in Bosnia and Herzegovina, which also showed that children were most afraid of the same three things [24]. Fear of injection in children is very common. The first thing a child that goes to a dentist is worried about is getting an injection [25]. Furthermore, this behavior of a child is strengthened, when he/she is threatened by their guardian or parents when resisting dental treatment [26]. Little research has been done about which age should a child get his or her first dental checkup. According to a few studies, the age of the child during his or her first visit is not of relevant importance, rather, the behavior of the dentist, and how comfortable the dentist makes the patient feel [27]. Other than the patient-doctor relationship, environmental factors play a huge role in either escalating dental anxiety or reducing it. The environmental factors were one of the main things addressed in this study. The private healthcare setting had four separate dental clinics, one for each patient, which consisted of a dentist and a dental assistant present in each one of them. Moreover, the walls of the clinic were painted with bright colors and had cartoon wallpapers on them, whereas in a public healthcare setting, a long hall with multiple dental units and multiple dentists, as well as dental assistants, were present at the same time. In this study, patients who attended a private dental healthcare setting had a higher prevalence of dental fear and anxiety. This was an astonishing finding since many factors, such as the number of people present in dental surgery and the general environment, should have contributed to a decrease in dental fear and anxiety compared to a public dental healthcare setting. A study done by Bares and Dundes indicated the importance of the appearance of a dental clinic in which they found that a clinic that has vibrant wall colors, a pleasant odor, and waiting rooms stocked with an ample number of magazines and books could significantly lower dental fear and anxiety [28]. A sensory-adapted dental environment has also shown evidence to reduce dental anxiety [29]. This concept aims to enhance the primary sense of sight, feel, touch, and smell centered on patient therapy. A study done by Shapiro et al. adopted this technique to reduce dental fear and anxiety, in which they used dimmed lighting, soothing music, and a special butterfly vest that hugs the child, which gives a relaxing pressure sensation. Although many of the social and environmental factors that reduced dental fear existed, some key factors were responsible for the difference seen in dental fear between the two healthcare settings. Public dental hospitals had low costs for dental treatments when compared to private dental hospitals. This was proportionate to the fact that families and children that visited a public dental hospital belonged to a low socioeconomic status. Few studies have linked that people who belong to a low socioeconomic status have a low prevalence of dental fear because they are less educated and aware of their oral health [30] and there have been some studies as well that indicate that people who belong to a higher socioeconomic status are generally more afraid [31]. This was consistent with our findings since most patients who visited a private dental hospital belonged to a higher socioeconomic status and, hence, were able to afford the cost of their dental procedures. Further research is needed to establish a strong correlation between low socioeconomic status and low dental fear. Another factor that increased dental fear in children visiting a private dental setting was the long wait times between appointments. Since there were only four dental surgeries and four dentists present at a time, this would cause a backlog in appointment times in which patients would have to wait weeks to get their next phase of treatment done. A study done by Bardy et al. found that a negative relationship existed between children’s dental attendance frequency and their anxiety [32]. The results of Bardy et al. were similar to a study done by Armfield et al., which stated that long time interval between dental visits negatively correlates with high levels of dental anxiety and irregular dental visits [33]. Since the ratio of patients to dentists in a private setup was extremely low, the time a dentist spent managing the child’s behavior before executing the procedure was reduced substantially. This in fact raised dental anxiety in a child. Studies have shown that a child’s first visit to a dentist is crucial in developing behavior toward dental treatment [34]. Research indicates that dentists' negative behavior, unfriendly dental staff, and shorter treatment times contribute to dental anxiety.

The strengths of this study were that, at the time of writing, this is the first study of its kind conducted in the Pakistani pediatric patient population comparing dental fear and anxiety in children across different healthcare settings, the use of a heavily validated tool (CFSS-DS) to measure dental fear and anxiety as well as individual variables on the tool that can be used for further analysis. We also used random sampling which has been known to limit sampling bias.

There are a few possible limitations to our study. One of the major limitations was that participants from only two hospitals were selected. Increasing the number of healthcare settings to conduct this study might show a different result due to an increase in sample size and alternate settings. The children completed the questionnaire in the presence of their parents which might have affected the questionnaire completion due to lack of privacy. It was difficult for children to comprehend and understand the five-point Likert scale and, hence, a more child-friendly scale such as one with a different range of colors or emoticons could be used which would remove the element of possible Hawthorne effect with respect to expressing their emotions. Furthermore, since this was a cross-sectional study, the causal inference could not be determined and confounding variables may have impacted our results.

Dental anxiety is a major problem in children that tip the balance between good and bad oral health. A more optimistic attitude toward dental treatments, ultimately resulting in good oral health, is needed to break this vicious cycle of dental fear.

## Conclusions

Dental fear and anxiety are common occurrences in children. CFSS-DS is a validated tool used to evaluate dental fear and anxiety in children. In our study, dental fear and anxiety were found to be significantly higher in the private dental setting when compared to the public dental setting which contradicted our initial hypothesis. Male children had significantly less fear when compared to female children. Irrespective of the setting, the children in our study demonstrated that they feared “choking” and “injections” more than the other discussed variables. There are several contributing factors to the causation of dental fear in children, and based on what we investigated there was no one specific factor that can be attributed to the cause of said fear alone. Dental fear and anxiety in children should be further researched to accurately determine and hope to eventually mitigate the causes of fear so that dental health checkups and procedures are more accessible and comfortable to the pediatric population to promote health equity in the younger population.
